# Diet as connecting factor: Functional brain connectivity in relation to food intake and sucrose tasting, assessed with resting‐state functional MRI in rats

**DOI:** 10.1002/jnr.24563

**Published:** 2019-11-26

**Authors:** Theresia J. M. Roelofs, Milou Straathof, Annette van der Toorn, Willem M. Otte, Roger A. H. Adan, Rick M. Dijkhuizen

**Affiliations:** ^1^ Department of Translational Neuroscience UMC Utrecht Brain Center University Medical Center Utrecht and Utrecht University Utrecht the Netherlands; ^2^ Biomedical MR Imaging and Spectroscopy Group Center for Image Sciences University Medical Center Utrecht and Utrecht University Utrecht the Netherlands; ^3^ Department of Child Neurology UMC Utrecht Brain Center University Medical Center Utrecht and Utrecht University Utrecht the Netherlands

**Keywords:** feeding behavior, magnetic resonance imaging, neuroimaging, rat brain

## Abstract

Eating disorders and obesity form a major health problem in Western Society. To be able to provide adequate treatment and prevention, it is necessary to understand the neural mechanisms underlying the development of eating disorders and obesity. Specific brain networks have been shown to be involved in feeding behavior. We therefore hypothesized that functional connectivity in neural networks involved in feeding behavior is dependent on the status of homeostatic energy balance, thus on being hungry or satiated. To test our hypothesis, we measured functional connectivity and amplitudes of neural signals within neural networks in relation to food intake and sucrose tasting in rats. Therefore, 16 male Wistar rats, of which eight were food‐restricted and eight were satiated, underwent resting‐state functional magnetic resonance imaging (rs‐fMRI) at 9.4 T. Subsequently, half of these animals underwent a sucrose tasting procedure followed by a second rs‐fMRI scan. Functional connectivity and amplitude of low‐frequency signal fluctuations were statistically analyzed in a linear mixed model. Although we did not detect a significant effect of food intake on functional connectivity before sucrose tasting, there was a trend toward interaction between group (satiated vs. hungry) and treatment (sucrose tasting). Functional connectivity between feeding‐related regions tended to decrease stronger upon sucrose tasting in satiated rats as compared to food‐restricted rats. Furthermore, rs‐fMRI signal amplitudes decreased stronger upon sucrose tasting in satiated rats, as compared to food‐restricted rats. These findings indicate that food intake and sucrose tasting can affect functional network organization, which may explain the specific patterns in feeding behavior.

## Graphical abstract

Food intake and sucrose tasting can affect functional network organization, as measured with resting‐state functional MRI in satiated and food‐restricted rats.

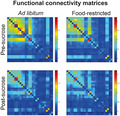

**Significance**

Our study demonstrates the potential of translational functional neuroimaging studies in rodents to elucidate how hunger, satiation and sweet taste affect functional connectivity between different brain regions. Our results show that these factors may affect functional networks in the brain, which may explain specific feeding behaviors.

## 1 INTRODUCTION

Disturbed regulation of feeding behavior and energy balance can lead to eating disorders and obesity. Obesity is a growing global health problem; most of the world's population lives in countries where more people die from overweight than from underweight (World Health Organisation, 2017). Even though obesity is preventable, its prevalence rises, which comes along with increasing risks of developing cardiovascular diseases, diabetes, musculoskeletal disorders and different types of cancer (World Health Organisation, 2017).

To combat obesity with adequate treatment and by prevention, it is necessary to get more insight into the exact neural processes and mechanisms of action underlying the development of overweight and obesity. Those neural processes involve multiple brain areas, associated with different aspects of the regulation of body weight, of which the role may vary between different states of homeostatic energy balance. These brain areas are functionally connected in neural networks, of which the organization may also differ depending on the status of homeostatic energy balance, thus on being hungry or satiated. An altered functional organization of these neural networks may result in different responses to food and food‐related cues, and could explain different feeding behaviors.

The organization of neural networks in relation to status of homeostatic energy balance has recently been investigated in several human neuroimaging studies. Wright and colleagues reported that fasting and satiation led to changes in functional connectivity of the insula and the hypothalamus as measured with resting‐state functional magnetic resonance imaging (rs‐fMRI) (Wright et al., 2016). The authors speculated that these regions form a network that regulates energy balance through cognitive control over eating. In another rs‐fMRI study, higher functional connectivity of the right supramarginal gyrus (as seed region) to the right midbrain, the bilateral midcingulate area, and the left hippocampus was measured when participants were in a hungry state as compared to a satiated state (Yousuf, Heldmann, Göttlich, Münte, & Doñamayor, 2017). According to the authors, the observed pattern of connectivity of the right supramarginal gyrus with the mesolimbic system in the hungry state could be related to the feeling of hunger, which is a very salient need that requires fulfilling and consequent recruitment of the mesolimbic system to drive motivational behavior. In line with these findings, Avery et al. reported a significant relationship between hunger status and functional connectivity of the left orbitofrontal cortex (OFC) to limbic regions (Avery et al., 2017). They found that functional connectivity between the left OFC and mid‐insula decreased when hunger deceased after a meal. However, this relationship was absent in obese participants.

Other studies failed to show the differences in functional connectivity between hungry and satiated healthy participants. Simon et al. (2017) and Al‐Zubaidi, Heldmann, Mertins, Jauch‐Chara, and Münte (2018) found no significant changes in functional connectivity as a result of hunger, although in the latter study the amplitudes of rs‐fMRI signals were increased in the posterior cingulate cortex and the anterior precuneus. A study that investigated the effect of weight loss on resting‐state functional connectivity in obese subjects also failed to detect a main effect of satiety on functional connectivity (Lepping et al., 2015).

The discrepancies in human rs‐fMRI studies on the relationship between status of homeostatic energy balance and neural network organization may be at least partly explained by a high level of methodological variation. To the best of our knowledge, there have been no studies that investigated the organization of neural networks in the resting brain in relation to food intake or tasting in animal models. Min and colleagues have successfully applied fMRI to map the brain regions that respond to intragastric infusion of nutrients (Min, Tuor, Koopmans, & Chelikani, 2011) or to gastric distention with an intragastric balloon (Min, Tuor, & Chelikani, 2011). However, resting‐state functional connectivity in large‐scale neural networks was not assessed in these studies. Therefore we set out to use an animal model to measure the functional network status in hungry and satiated subjects in a tightly controlled and highly standardized experimental setup. Furthermore, this setup would also allow the assessment of the influence of sucrose tasting without interference of the cognitive component associated with the differences in the level of satiation. To this end, we performed rs‐fMRI in anesthetized food‐restricted or satiated rats, before and after sucrose tasting. We hypothesized that functional connectivity between areas involved in feeding behavior and areas involved in reward and motivation would be higher when animals are hungry, and that this functional connectivity would decline in response to sucrose tasting.

## 2 MATERIALS AND METHODS

### 2.1 Animals

Experiments were approved by the Animal Ethics Committee of the University Medical Center Utrecht, the Netherlands, and were conducted in agreement with Dutch laws (“Wet op de Dierproeven”, 1996) and European regulations (Guideline 86/609/EEC).

**Figure 1:**
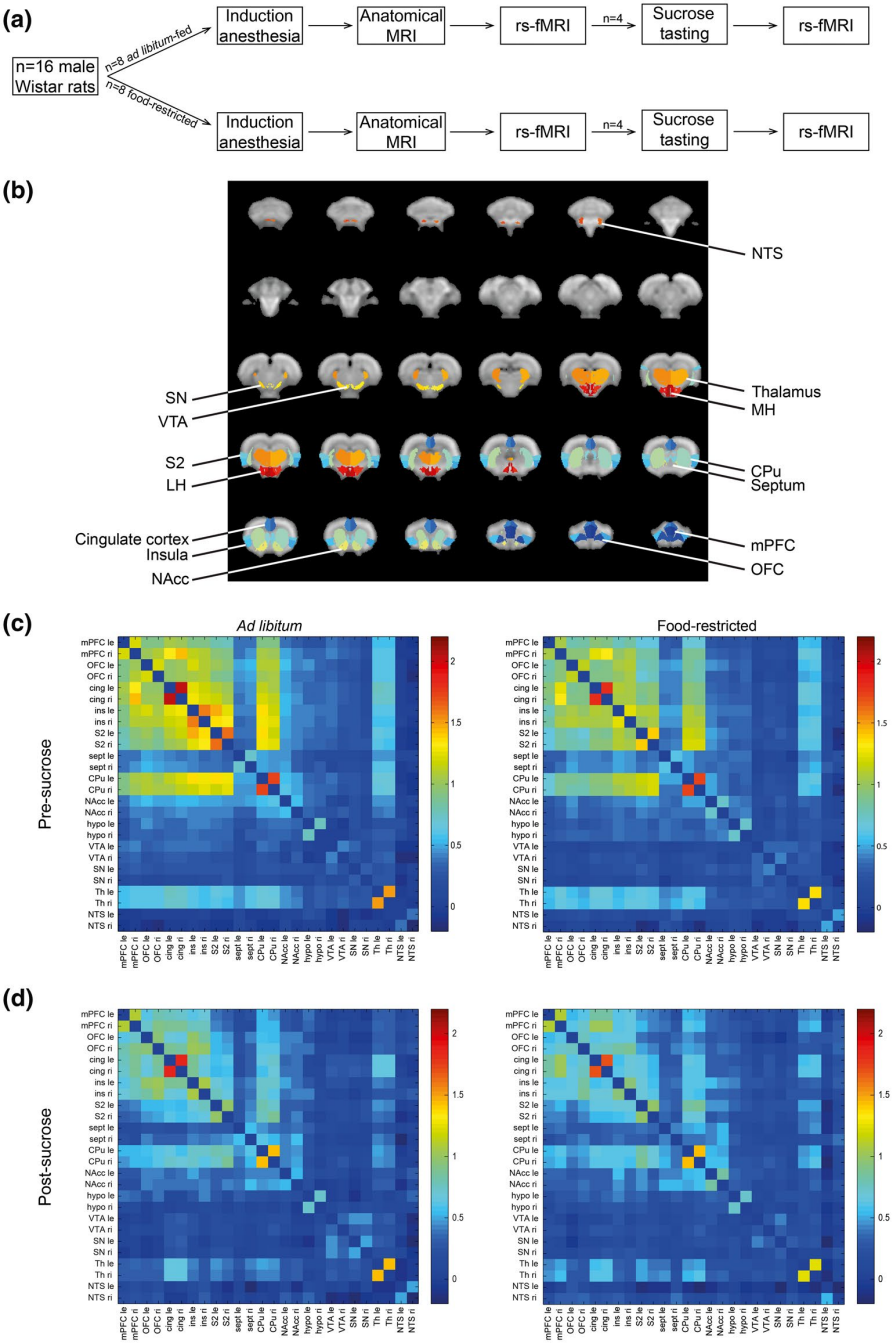
Novel experimental resting‐state functional magnetic resonance imaging (fMRI) approach allows detection of alterations in functional connectivity as a result of food intake and sucrose tasting. (a) Experimental outline. (b) Regions of interest (ROIs) as used in the study. (c) Inter‐ and intrahemispheric functional connectivity between different ROI combinations assessed in *ad libitum*‐fed (left) and food‐restricted rats (right) prior to sucrose tasting. Functional connectivity is shown as Fisher‐transformed *z*′ of Pearson correlation coefficient *r*. (d) Inter‐ and intrahemispheric functional connectivity between different ROI combinations assessed in *ad libitum*‐fed (left) and food‐restricted rats (right) after sucrose tasting. cing, cingulate cortex; CPu, caudate putamen; hypo, hypothalamus; ins, insula; le, left; LH, lateral hypothalamus; MH, medial hypothalamus; mPFC, medial prefrontal cortex; NAcc, nucleus accumbens; NTS, nucleus of the solitary tract; OFC, orbitofrontal cortex; ri, right; S2, secondary somatosensory cortex; sept, septum; SN, substantia nigra; Th, thalamus; VTA, ventral tegmental area

We used 16 adult male Wistar rats (Crl:WU, Charles River, Sulzfeld, Germany) with a mean (± standard deviation) body weight of 240 (±6) g upon arrival. Rats were housed individually under controlled temperature and humidity conditions, and under a 12 hr light/dark cycle (lights on at 7:00 a.m.). Animals had *ad libitum* access to water, and a perspex tube was provided as cage enrichment. Rats were randomly assigned to two equally sized groups. One group of rats (*n* = 8) had *ad libitum* access to chow, the other group (*n* = 8) was food‐restricted (Figure 1a). Food restriction started 1 week prior to scanning and involved a diet of 10 g of chow per day till the animal reached 90% of its initial bodyweight. When this point was reached before the actual day of scanning, animals received the amount of chow needed to maintain this bodyweight (which differed per rat, but was always between 10 and 20 g of chow). The experimenter could not be blinded to group assignment, since animal caretaking and MRI scanning were done by the same person. Rats’ mean (± standard deviation) body weight at time of scanning was 324 (±40) g.

### 2.2 *In vivo* MRI acquisition

We conducted *in vivo* MRI measurements using a 9.4 T horizontal bore Magnetic Resonance (MR) system equipped with a 100 mT/m gradient coil (Agilent). We used a home‐built 90 mm diameter Helmholtz volume coil for signal excitation, and an inductively coupled 25 mm diameter surface coil for signal reception. Anesthesia was induced with 3%–5% isoflurane in O_2_/air (1:4), and animals were endotracheally intubated for mechanical ventilation. For fMRI‐based detection of activation in response to sucrose tasting, two Protex polyethylene tubes were placed above the tongue. The polyethylene tubes enabled sucrose delivery and water rinsing while the animal was inside the MR scanner.

After animals were positioned inside the scanner, anesthesia was reduced to 1.5% isoflurane in O_2_/air (1:4). We maintained anesthesia at 1.5% during the entire MRI protocol. End‐tidal CO_2_ was monitored with a capnograph (Microcap, Oridion Medical 1987 Ltd., Jerusalem, Israel). Body temperature was maintained at 37.0 ± 1.0°C.

We first acquired anatomical images using a balanced steady‐state free precession sequence, with four phase cycling angles (0°, 90°, 180°, and 270°), repetition time (TR)/echo time (TE) = 5/2.5 ms, flip angle = 20°, field‐of‐view (FOV) = 40 × 32 × 24 mm, and matrix size = 160 × 128 × 96 voxels (scan time: 10 min). The resulting spatial resolution of these anatomical images was 250 μm in all directions.

Next, we conducted rs‐fMRI, for which we used a 3D gradient echo planar imaging (EPI) sequence. The read‐out and first phase‐encode dimensions were covered in a single‐shot EPI, while linear phase encoding was used for the second phase‐encode dimension. We acquired 800 images with an acquisition time of 862.4 ms per volume (total scan time: 11 min and 30 s), and TR/TE = 30.8/16 ms, flip angle = 13°, FOV = 36 × 36 × 16.8 mm, matrix size = 60 × 60 × 28, and thus an isotropic spatial resolution of 600 µm.

After the rs‐fMRI scan, half of the animals (*n* = 4 in both the food‐restricted and *ad libitum*‐fed groups) underwent fMRI during sucrose tasting, which consisted of five repetitions of sucrose application on the tongue. After sucrose tasting, a second rs‐fMRI scan was performed (Figure 1a).

### 2.3 MRI data processing and analysis

#### 2.3.1 Anatomical MRI data

Non‐uniformity correction was performed on anatomical images using *n3*, and brain masks were obtained by applying the *Brain Extraction Tool* to the anatomical images, both as provided by *FSL* (FMRIB's Software Library, www.fmrib.ox.ac.uk/fsl, version 5.0.9). The individual anatomical images were masked and registered to an anatomical MRI template that was matched to a 3D model of a rat brain atlas (Paxinos & Watson, 2007), using the affine intermodal image registration tool *FLIRT* (FMRIBs Linear Image Registration Tool, v6.0). Co‐registered anatomical images were averaged to acquire an anatomical template in atlas space. The original individual anatomical images were registered to this anatomical template using *FLIRT* followed by *FNIRT* (FMRIBs Nonlinear Image Registration Tool). Inverse coefficients were calculated to register regions of interest (ROIs) from atlas space to individual anatomical space. ROIs extracted from the rat brain atlas (Paxinos & Watson, 2007) consisted of the left and right ventral tegmental area (VTA), nucleus accumbens (NAcc), insula, medial hypothalamus (MH), lateral hypothalamus (LH), medial prefrontal cortex (mPFC), OFC, caudate putamen (CPu), and nucleus of the solitary tract (NTS) (Figure 1b).

#### 2.3.2 Resting‐state fMRI data processing

All rs‐fMRI images were corrected for subject motion using *MCFLIRT*, and image intensity non‐uniformity correction was performed using *n3* (solely for registration purposes). Using *FLIRT*, rs‐fMRI images were registered to a mean rs‐fMRI image of a single representative rat. Co‐registered images were averaged to create an rs‐fMRI template specific for this batch of animals. To exclude tissue outside the brain, brain masks were obtained by applying the *Brain Extraction Tool* from *FSL* to the template. Since this tool is difficult to apply to rodent resting‐state data, the brain mask for the rs‐fMRI template was somewhat increased in size, resulting in inclusion of some non‐brain voxels. To obtain a more accurate brain mask, the initial brain mask was registered to individual space using *FLIRT*, and the mean image of each rat's rs‐fMRI scan was masked using this initial mask. The individual masked mean rs‐fMRI images were subsequently registered into Waxholm atlas space (Papp, Leergaard, Calabrese, Johnson, & Bjaalie, 2014) using *FLIRT*, and a mean of these registered images was calculated to create a reference image in atlas space. Using *FNIRT*, the masked individual mean rs‐fMRI images were nonlinearly registered to this reference image, and inverse coefficients were calculated to register the optimized masks from atlas space to individual fMRI space. These optimized masks were obtained by averaging the co‐registered individual images in reference space, and subsequently thresholding this average image to exclude all non‐brain voxels. The resulting masks optimally delineated the brain and were registered into individual space using the previously calculated coefficients. The ROIs were registered into individual space using the same coefficients. The individual rs‐fMRI data as well as the ROIs in individual space were masked using the optimized final masks.

#### 2.3.3 Resting‐state fMRI data analysis—Whole‐brain connectivity maps

Rs‐fMRI data were band‐pass filtered between 0.01 and 0.1 Hz using 3dFourier as provided by *AFNI* (Analysis of Functional NeuroImages, Version Debian‐16.2.07, https://afni.nimh.nih.gov/). Temporal signal‐to‐noise ratio (tSNR) was calculated per individual animal using *FSLMATHS* as implemented in *FSL*. The resulting tSNR map was used to mask out voxels with tSNR < 10 and this mask was applied to the 4D filtered rs‐fMRI data and to the registered ROIs. This resulted in occasional exclusion of individual voxels from ROIs. Functional connectivity maps displaying the Fisher‐transformed *z*′ of Pearson correlation coefficient *r* were calculated using *fcmap* as provided by *FSL*, with the left or right VTA, NAcc, insula, MH, LH, mPFC, OFC, CPu, or NTS as seed ROI. These individual functional connectivity maps were registered to the reference image in atlas space, in order to perform group analysis. Mean seed‐to‐whole‐brain functional connectivity maps were calculated for food‐restricted animals and for *ad libitum*‐fed animals using *FSLMATHS*.

Mean functional connectivity maps as described above were also calculated after animals tasted sucrose.

#### 2.3.4 Resting‐state fMRI data analysis—ROI analyses

Functional connectivity matrices were obtained from the Fisher‐transformed *z*′ of Pearson correlation coefficients *r* between ROIs using *FSL* and MATLAB (The MathWorks Inc., version R2014a). Average functional connectivities between specific ROIs were compared between the different groups. We assessed functional connectivity between homologous ROIs in both hemispheres (interhemispheric functional connectivity) as well as between ROIs within hemispheres (intrahemispheric functional connectivity). To statistically compare functional connectivity values and signal amplitude values on a group level in the pre‐sucrose data, we performed a linear mixed model analysis within the *nlme* package (Pinheiro, Bates, DebRoy, Sarkar, & R core team, 2018) with the factor group (*ad libitum* or food‐restricted) as between‐subject variable and with correction for multiple comparisons. For the pre‐ and post‐sucrose tasting data combined, we performed a linear mixed model analysis with correction for multiple comparisons within the *nlme* package (Pinheiro et al., 2018) with the factor group (*ad libitum* or food‐restricted) as between‐subject variable and the factor time (pre‐ and post‐sucrose tasting) as within‐subject variable. In all mixed models, random effects were modelled hierarchically with the factor ROI (or connection) nested within the factor rat, to correct for multiple connections measured within the same animal. Examples of the linear mixed model software code used for the current analyses as well as data acquired in this study are available online (https://github.com/wmotte/fcfood). Comparison of functional connectivities (intra‐ and interhemispherically) between groups was done for ROIs that are known to be involved in the regulation of energy balance and feeding behavior. These ROIs included the VTA, NAcc, insula, MH, LH, mPFC, OFC, CPu, and NTS (Figure 1b). This was done for pre‐sucrose tasting data to assess the influence of status of homeostatic energy balance and for post‐sucrose tasting data to assess the influence of status of homeostatic energy balance after sucrose tasting.

#### 2.3.5 Resting‐state fMRI data analysis—Fractional amplitude of low‐frequency fluctuations

As a measure of the magnitude of brain activity, we assessed the fractional amplitude of low‐frequency fluctuations (fALFFs), calculated from the rs‐fMRI BOLD signals as the ratio of the power of each frequency in the low‐frequency range (0.01–0.1 Hz) to that of the entire frequency range (Al‐Zubaidi et al., 2018; Zou et al., 2008). The fALFF has been shown to be more sensitive than ALFF and also corrects for physiological noise (Zou et al., 2008). We transformed the time series of the unfiltered motion‐corrected rsfMRI data to a frequency domain using *fslpspec* as provided by *FSL*. The rsfMRI data were temporally band‐pass filtered (0.01 < Hz < 0.1). Power spectra were calculated in *fslpspec*. The resulting 4D power spectra of filtered and unfiltered data were used to calculate cumulative 3D power spectra per rat using *FSLMATHS*. The cumulative power spectra from the filtered data were divided by the cumulative power spectra of the unfiltered data to achieve fALFF maps for each rat. Individual fALFF maps were registered to the resting‐state reference image in atlas space using the coefficients described earlier. We compared mean fALFF maps between the groups (*ad libitum*‐fed vs. food‐restricted groups) by calculating the mean of all fALFF maps per group. Using a second level generalized linear model (GLM)‐based analysis followed by a false discovery rate (FDR) correction for multiple comparisons, we compared mean fALFF maps between the two groups. This was done for pre‐ as well as post‐sucrose tasting data.

In addition to the whole‐brain fALFF analysis, we compared fALFF between *ad libitum*‐fed and food‐restricted animals in a feeding behavior‐related network comprising the following ROIs: VTA, NAcc, insula, MH, LH, mPFC, OFC, CPu, and NTS. This was done for pre‐sucrose tasting data to assess the influence of status of homeostatic energy balance on: fALFF; post‐sucrose tasting data to assess the influence of status of homeostatic energy balance on fALFF after sucrose tasting; and the sucrose tasting‐induced differences in fALFF.

## 3 RESULTS

Three animals (one from the food‐restricted group that also underwent sucrose tasting; one from the *ad libitum‐*fed group that also underwent sucrose tasting; and one animal from the food‐restricted group that did not underwent sucrose tasting) had to be excluded from the study due to significant MRI artefacts. The final group sizes for pre‐sucrose tasting data were *n* = 6 for the food‐restricted group, and *n* = 7 for the *ad libitum* group. Final group sizes for post‐sucrose tasting data were *n* = 3 for the food‐restricted and *ad libitum‐*fed groups.

### 3.1 A general decrease in functional connectivity upon food restriction

Functional connectivities between different ROIs in the food‐restricted and *ad libitum*‐fed groups prior to sucrose tasting are displayed as connectivity matrices in Figure 1c. Functional connectivity was clearly stronger between anterior cortical regions, such as the mPFC, OFC, cingulate cortex, CPu, insula, and S2, than between more posterior regions, such as the NAcc, hypothalamus, VTA, and SN. The functional connectivity matrices displayed a generally lower functional connectivity in the food‐restricted group. After sucrose tasting, functional connectivities were generally lower (Figure 1d).

**Figure 2:**
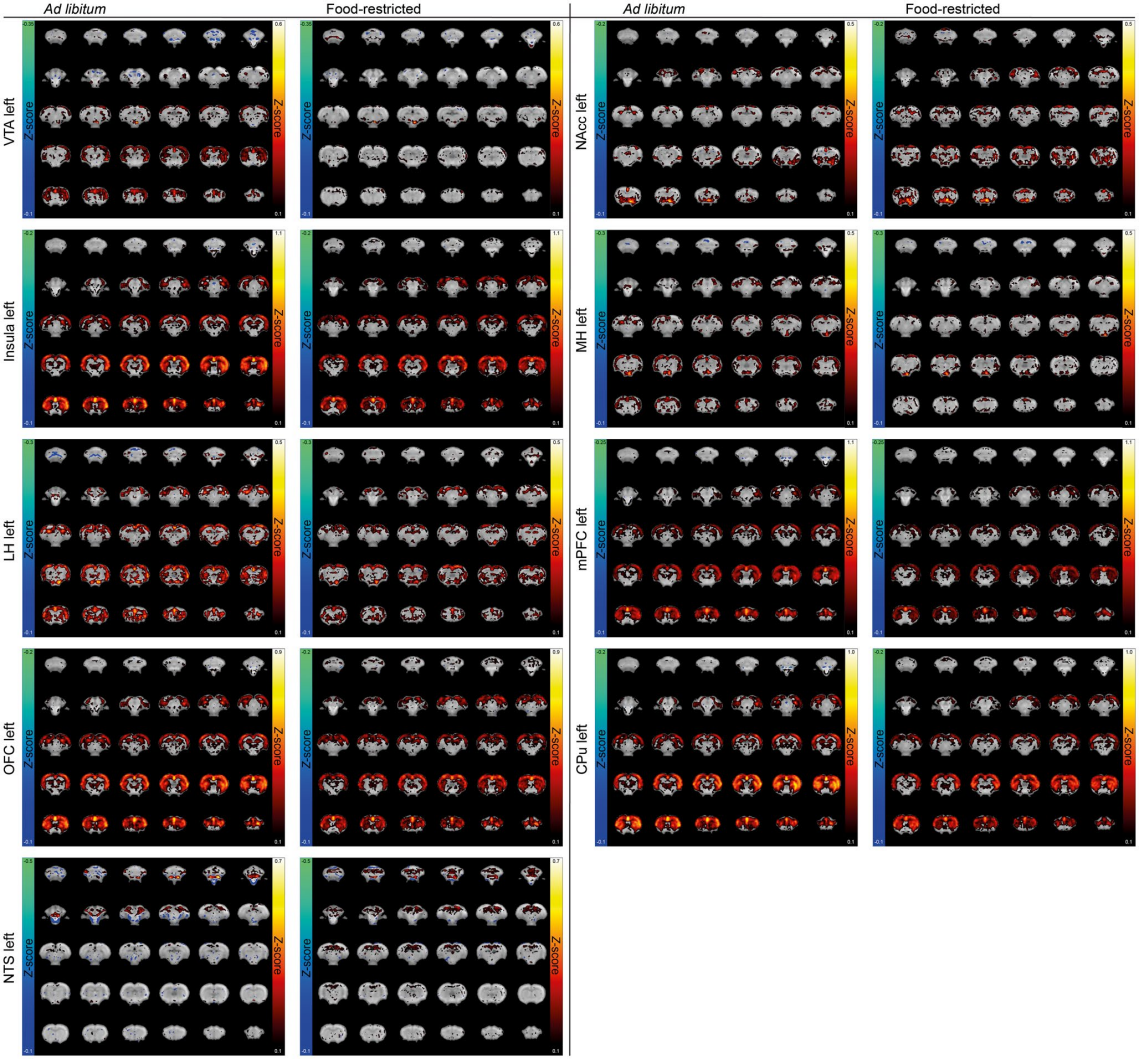
Food intake‐dependent functional connectivity maps with different seed regions. Mean functional connectivity maps were obtained by calculation of the Fisher‐transformed *z*′ of Pearson correlation coefficient *r* and display functional connectivity for *ad libitum‐*fed rats (left) and food‐restricted rats (right) prior to sucrose tasting. Different left ROIs were used as seed regions: CPu, caudate putamen; LH, lateral hypothalamus; MH, medial hypothalamus; mPFC, medial prefrontal cortex; NAcc, nucleus accumbens; NTS, nucleus of the solitary tract; OFC, orbitofrontal cortex; VTA, ventral tegmental area

**Figure 3:**
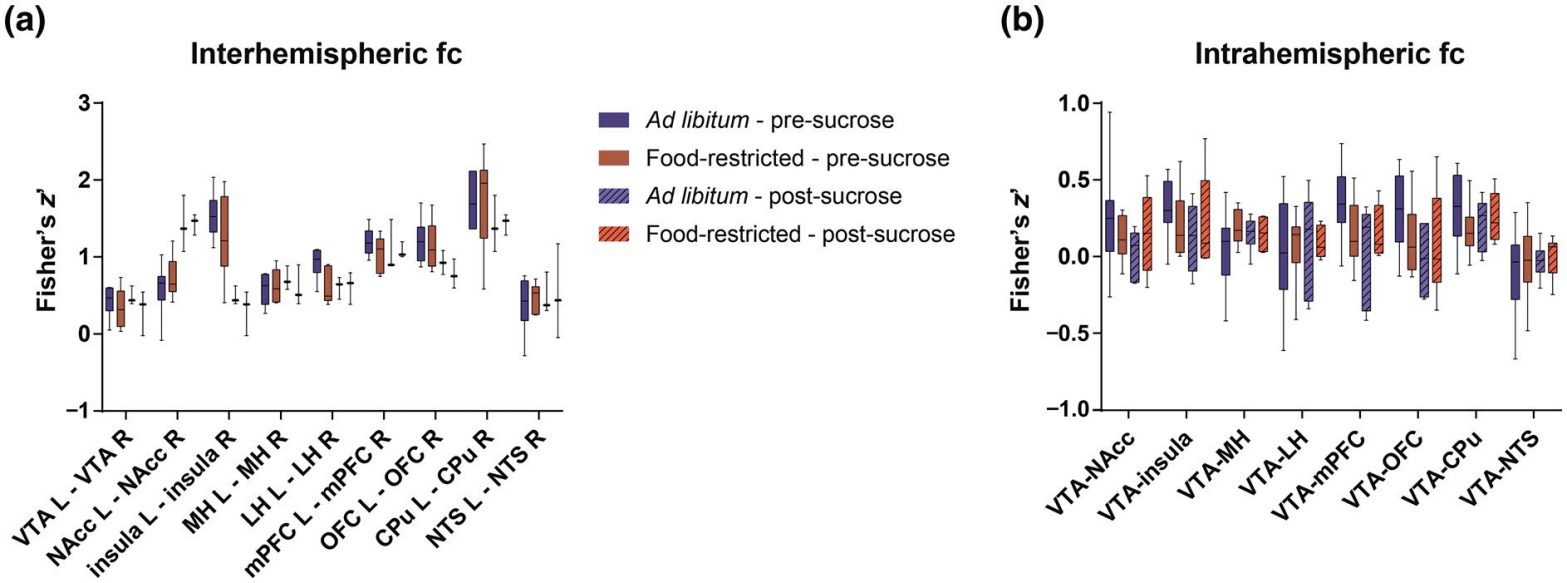
Inter‐ and intrahemispheric functional connectivity in food‐restricted and *ad libitum‐*fed rats before and after sucrose tasting. (a) Functional connectivity between homologous ROIs, interhemispheric functional connectivity, and (b) functional connectivity between the left VTA and left ROI plus the right VTA and the right ROI, intrahemispheric functional connectivity, compared between *ad libitum*‐fed and food‐restricted animals pre‐ and post‐sucrose tasting. Boxplots represent the minimum, first quartile, median, third quartile, and maximum functional connectivity values. No significant differences were detected in pre‐sucrose tasting data. After sucrose tasting, interhemispheric functional connectivities tended to be lower in the *ad libitum‐*fed group compared to the food‐restricted group. Fc, functional connectivity; L, left; R, right; CPu, caudate putamen; LH, lateral hypothalamus; MH, medial hypothalamus; mPFC, medial prefrontal cortex; NAcc, nucleus accumbens; NTS, nucleus of the solitary tract; OFC, orbitofrontal cortex; VTA, ventral tegmental area

Seed‐based functional connectivity maps for different unilateral ROIs before sucrose tasting are shown in Figure 2 (for left ROIs as seed region) and Supporting Information Figure S1 (for right ROIs as seed region). In general, these functional connectivity maps revealed strong interhemispheric connectivity between homologous areas, and strong connectivity with cortical areas. A comparable pattern was observed after sucrose tasting (Supporting Information Figure S2). Comparison of seed‐based functional connectivity patterns between *ad libitum*‐fed and food‐restricted rats before sucrose tasting revealed a generally stronger functional connectivity for all ROIs in the *ad libitum‐*fed group prior to sucrose tasting. This is also apparent in Figure 3, which shows the interhemispheric functional connectivity between homologous ROIs (Figure 3a), and the intrahemispheric functional connectivity between the VTA and the other ROIs (Figure 3b). At group level, we found no significant difference between *ad libitum*‐fed and food‐restricted animals before sucrose tasting (group level statistics for interhemispheric functional connectivity: estimate, −0.067; 95% CI, −0.287–0.154; *p* = 0.520; for intrahemispheric functional connectivity: estimate, 0.061; 95% CI, −0.223–0.101; *p* = 0.423). However, there was a trend for interaction between group and time for intrahemispheric connections (group level statistics: estimate, −0.095; 95% CI, −0.199–0.010; *p* = 0.077).

### 3.2 Difference in fALFF between satiated and food‐restricted rats

**Figure 4:**
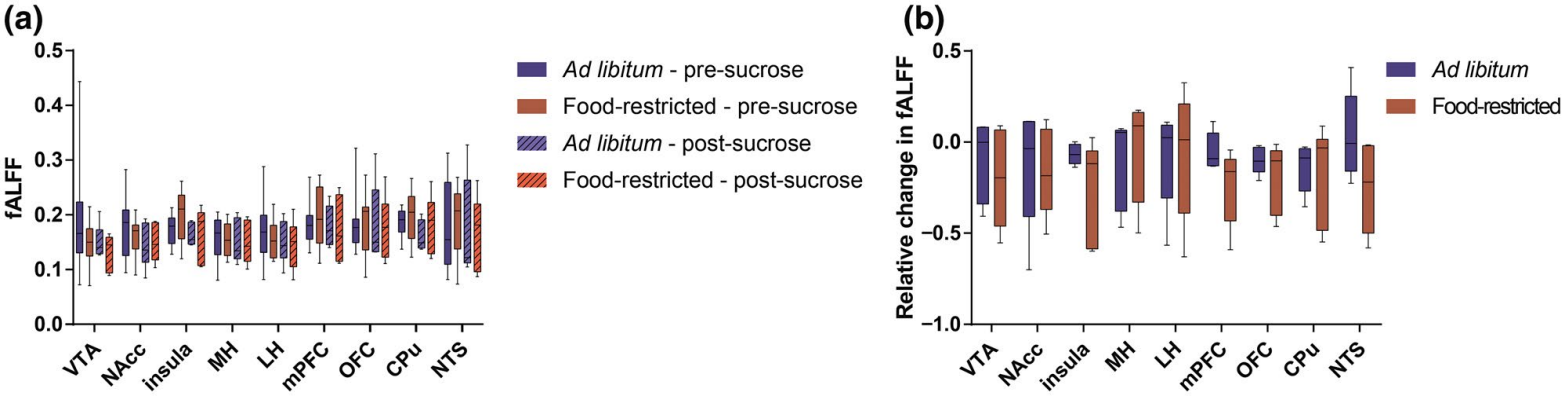
Fractional amplitude of low‐frequency fluctuations (fALFFs) in satiated and food‐restricted rats before and after sucrose tasting. (a) fALFF in different ROIs was significantly different between pre‐ and post‐sucrose tasting, probably explained by a decrease in fALFF upon sucrose tasting. There was also a significant group and time interaction effect at the ROI level. (b) The relative difference in fALFF of post‐ as compared to pre‐sucrose data, compared between *ad libitum‐*fed and food‐restricted rats, did not reveal significant differences. Boxplots represent the minimum, first quartile, median, third quartile, and maximum fALFF values. CPu, caudate putamen; LH, lateral hypothalamus; MH, medial hypothalamus; mPFC, medial prefrontal cortex; NAcc, nucleus accumbens; NTS, nucleus of the solitary tract; OFC, orbitofrontal cortex; VTA, ventral tegmental area

Mean fALFF maps are shown in Supporting Information Figure S3. We detected a significant effect of time (sucrose tasting) on fALFF at the ROI level, as well as a significant interaction effect between group and time. The time effect (group level statistics: estimate, 0.0191; 95% CI, 0.0072–0.0311, *p* = 0.002) probably reflects a decrease in fALFF upon sucrose tasting (see Figure 4), whereas the group and time interaction effect at the ROI level (group level statistics: estimate, 0.0209; 95% CI, 0.0040–0.0378; *p* = 0.016) might be related to an overall stronger decrease in fALFF upon sucrose tasting in food‐restricted animals as compared to *ad libitum*‐fed animals (Figure 4b).

## 4 DISCUSSION

In this study we explored whether status of homeostatic energy balance, based on food intake, and sucrose tasting are reflected in resting‐state functional connectivity and amplitudes of rs‐fMRI signals within neural networks involved in feeding behavior. With a recently developed protocol, we acquired rs‐fMRI scans in food‐restricted and *ad libitum‐*fed rats before and after sucrose tasting, similar to paradigms used in human neuroimaging studies on the regulation of feeding behavior. Our data revealed a trend toward higher functional connectivity in *ad libitum‐*fed rats compared to food‐restricted rats. Analysis of the fractional amplitude of the low‐frequency resting‐state signals suggested a decrease in fALFF in response to sucrose tasting.

The observed functional connectivity between the insula, cingulate cortex, and frontal cortices in rats agrees with studies in humans that have revealed a resting‐state network linked to limbic brain regions (Al‐Zubaidi et al., 2018; Zuo et al., 2010). The anterior insula contains the primary taste cortex and is involved in processing gustatory stimulation, but also in processing interoceptive signals from the gut (de Araujo & Rolls, 2004; Gagnon, Kupers, & Ptito, 2015; Small, 2010; Small et al., 2003). Limbic regions such as the VTA and the SN are involved in reward and motivational behavior, and are as such also involved in feeding behavior (Kelley, Baldo, Pratt, & Will, 2005). In the current study, we detected higher functional connectivity of cortical areas, such as the insula, mPFC, and cingulate cortex to these limbic structures in *ad libitum‐*fed rats as compared to food‐restricted rats. This is in contrast to our hypothesis, since we expected higher functional connectivity between areas involved in feeding behavior, reward, and motivation in hungry animals, in line with some human fMRI studies that reported higher functional connectivity in hungry as compared to satiated subjects (Avery et al., 2017; Yousuf et al., 2017). On the other hand, Wright and colleagues found decreases as well as increases in functional connectivity upon fasting (Wright et al., 2016), and other studies failed to show an effect of satiety condition on functional connectivity (Al‐Zubaidi et al., 2018; Lepping et al., 2015; Simon et al., 2017).

Our linear mixed model analysis revealed a trend toward an interaction between food intake and sucrose tasting. This indicates that hunger status may have an effect on how neural networks respond to a feeding‐related stimulus. Others have shown that resting‐state neural signals are affected by meal consumption or glucose administration (Al‐Zubaidi et al., 2018; De Silva, Salem, Matthews, & Dhillo, 2012). However, our results should be interpreted with caution, since the number of animals per group was low in the sucrose tasting experiment.

The fALFF has previously been suggested as a sensitive marker to assess homeostatic changes in the brain (Al‐Zubaidi et al., 2018). We detected a significant decrease in fALFF upon sucrose tasting in both groups, as well as an interaction effect. This could potentially be related to a parallel decrease in functional connectivity upon sucrose tasting.

A limitation of the current study is the small sample size. Especially the post‐sucrose tasting data should be interpreted with caution, since both the food‐restricted and the *ad libitum*‐fed groups consisted of only three rats after sucrose tasting. In addition, we were not able to scan animals during actual feeding behavior under awake conditions. Although sucrose tasting mimics an aspect of the natural process of satiation, we cannot exclude the potential differences in the effects on neural network signaling.

In conclusion, our fMRI experiments in rats show that food intake and sucrose tasting can lead to specific changes in the connectivity and activity of neural networks involved in feeding behaviors. The applied paradigms are comparable to those used in human neuroimaging studies, reflecting its translational value. Our proof‐of‐principle study could provide a starting point for future studies to further disentangle the complex interaction between (changes in) status of homeostatic energy balance and brain signaling, which can aid in elucidation of behavioral phenotypes of feeding in health and disease. Such studies could include the assessment of possible variations in neural network organization between lean and obese rats, which have a differently regulated status of energy balance.

## DECLARATION OF TRANSPARENCY

The authors, reviewers, and editors affirm that in accordance with the policies set by the *Journal of Neuroscience Research* this manuscript presents an accurate and transparent account of the study being reported and that all critical details describing the methods and results are present.

## CONFLICT OF INTEREST

The authors declare no conflict of interest of any type.

## AUTHOR CONTRIBUTIONS

*Conceptualization*, T.J.M.R., R.A.H.A., R.M.D.; *Methodology*, T.J.M.R., A.v.d.T., R.A.H.A., R.M.D.; *Software*, A.v.d.T., W.M.O.; *Validation*, T.J.M.R., M.S.; *Investigation*, T.J.M.R.; *Formal Analysis*, T.J.M.R., M.S., W.M.O.; *Resources*, T.J.M.R., A.v.d.T., W.M.O., R.A.H.A., R.M.D.; *Data Curation*, T.J.M.R.; *Writing – Original Draft*, T.J.M.R.; *Writing – Review & Editing*, T.J.M.R., M.S., R.A.H.A., R.M.D.; *Visualization*, T.J.M.R.; *Supervision*, R.A.H.A., R.M.D.; *Project Administration*, T.J.M.R.; *Funding Acquisition*, R.A.H.A., R.M.D..

## Supporting information


**Figure S1**. Functional connectivity maps with different right ROIs as seed regions from ad libitum‐fed and food‐restricted rats. Mean functional connectivity maps were obtained by calculation of the Fisher‐transformed *z*′ of Pearson correlation coefficient *r* and display functional connectivity for ad libitum‐fed rats (left) and food‐restricted rats (right). Different right ROIs were used as seed regions: CPu, caudate putamen; LH, lateral hypothalamus; MH, medial hypothalamus; mPFC, medial prefrontal cortex; NAcc, nucleus accumbens; NTS, nucleus of the solitary tract; OFC, orbitofrontal cortex; VTA, ventral tegmental area
**Figure S2**. Functional connectivity maps after sucrose tasting with different seed regions from ad libitum‐fed and food‐restricted rats. Mean functional connectivity maps were obtained by calculation of the Fisher‐transformed *z*′ of Pearson correlation coefficient r and show functional connectivity for ad libitum‐fed (left) and food‐restricted rats (right) post‐sucrose tasting. Different left ROIs were used as seed regions: CPu, caudate putamen; LH, lateral hypothalamus; MH, medial hypothalamus; mPFC, medial prefrontal cortex; NAcc, nucleus accumbens; NTS, nucleus of the solitary tract; OFC, orbitofrontal cortex; VTA, ventral tegmental area
**Figure S3**. Mean fractional amplitude of low‐frequency fluctuations (fALFFs) maps in different states of energy balance. (a) Whole‐brain fALFF pre‐sucrose tasting in ad libitum‐fed rats. (b) Whole‐brain fALFF pre‐sucrose tasting in food‐restricted rats. (c) Whole‐brain fALFF post‐sucrose tasting in ad libitum‐fed rats. (d) Whole‐brain fALFF post‐sucrose tasting in food‐restricted rats

Transparent Science Questionnaire for Authors

Transparent Peer Review Report

## Data Availability

The data that support the findings of this study are available from the corresponding author upon reasonable request.
