# Supplementary material for: Diet as connecting factor: Functional brain connectivity in relation to food intake and sucrose tasting, assessed with resting‐state functional MRI in rats
Source: J Neurosci Res. 2019 Nov 26;100(5):1182–90. doi: 10.1002/jnr.24563 (PMC9291979; doi:10.1002/jnr.24563)
Supplement: Supplementary file 1 — Figure S1. Functional connectivity maps with different right ROIs as seed regions from ad libitum‐fed and food‐restricted rats. Mean functional connectivity maps were obtained by calculation of the Fisher‐transformed z′ of Pearson correlation coefficient r and display functional connectivity for ad libitum‐fed rats (left) and food‐restricted rats (right). Different right ROIs were used as seed regions: CPu, caudate putamen; LH, lateral hypothalamus; MH, medial hypothalamus; mPFC, medial prefrontal cortex; NAcc, nucleus accumbens; NTS, nucleus of the solitary tract; OFC, orbitofrontal cortex; VTA, ventral tegmental area Figure S2. Functional connectivity maps after sucrose tasting with different seed regions from ad libitum‐fed and food‐restricted rats. Mean functional connectivity maps were obtained by calculation of the Fisher‐transformed z′ of Pearson correlation coefficient r and show functional connectivity for ad libitum‐fed (left) and food‐restricted rats (right) post‐sucrose tasting. Different left ROIs were used as seed regions: CPu, caudate putamen; LH, lateral hypothalamus; MH, medial hypothalamus; mPFC, medial prefrontal cortex; NAcc, nucleus accumbens; NTS, nucleus of the solitary tract; OFC, orbitofrontal cortex; VTA, ventral tegmental area Figure S3. Mean fractional amplitude of low‐frequency fluctuations (fALFFs) maps in different states of energy balance. (a) Whole‐brain fALFF pre‐sucrose tasting in ad libitum‐fed rats. (b) Whole‐brain fALFF pre‐sucrose tasting in food‐restricted rats. (c) Whole‐brain fALFF post‐sucrose tasting in ad libitum‐fed rats. (d) Whole‐brain fALFF post‐sucrose tasting in food‐restricted rats [file JNR-100-1182-s003.pdf]

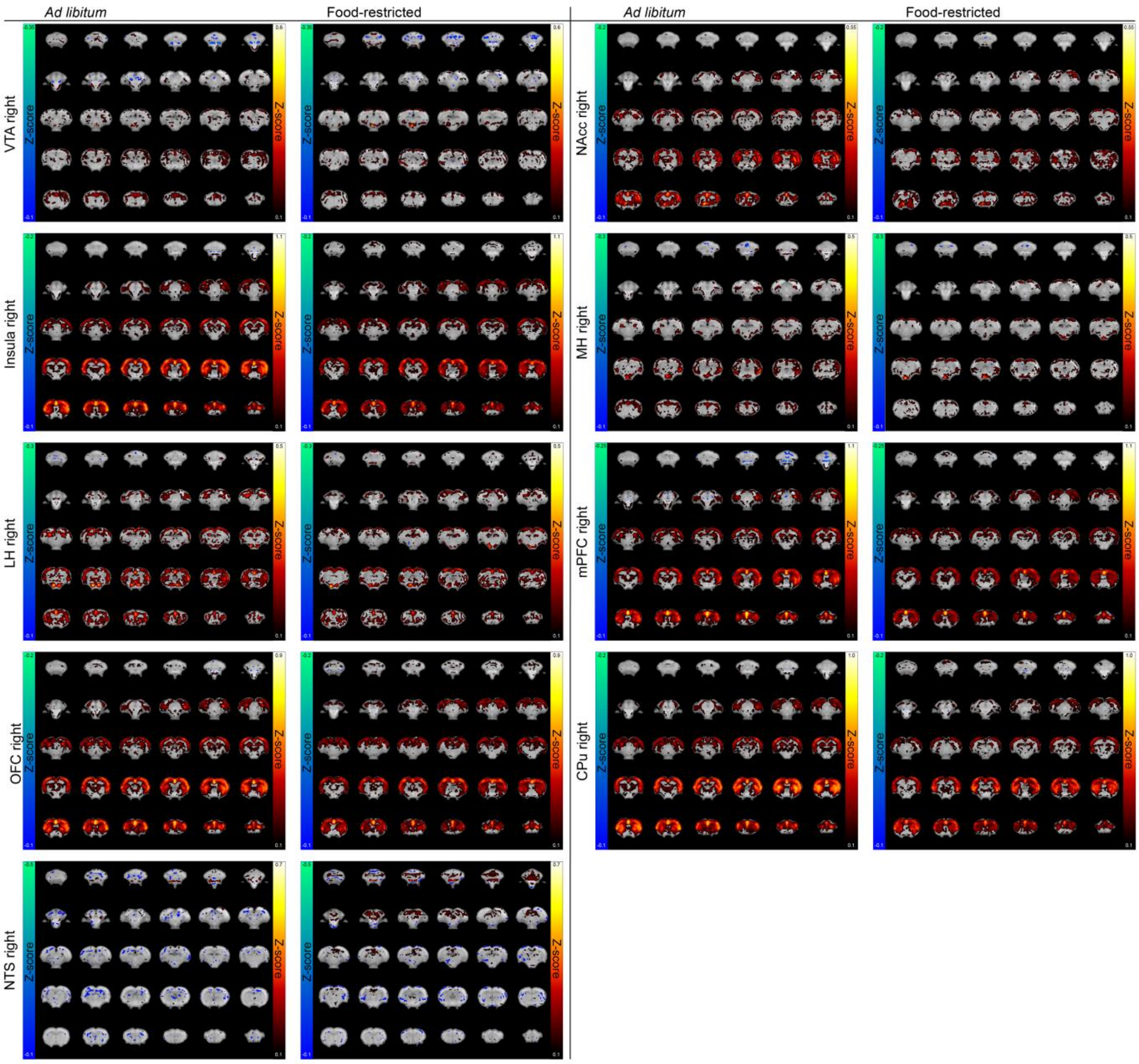

Supplementary Figure 1. Functional connectivity maps with different right ROIs as seed regions from *ad libitum*-fed and food-restricted rats. Mean functional connectivity maps were obtained by calculation of the Fisher-transformed z' of Pearson correlation coefficient *r* and display functional connectivity for *ad libitum*-fed rats (left) and food-restricted rats (right). Different right ROIs were used as seed regions: CPu, caudate putamen; LH, lateral hypothalamus; MH, medial hypothalamus; mPFC, medial prefrontal cortex; NAcc, nucleus accumbens; NTS, nucleus of the solitary tract; OFC, orbitofrontal cortex; VTA, ventral tegmental area.

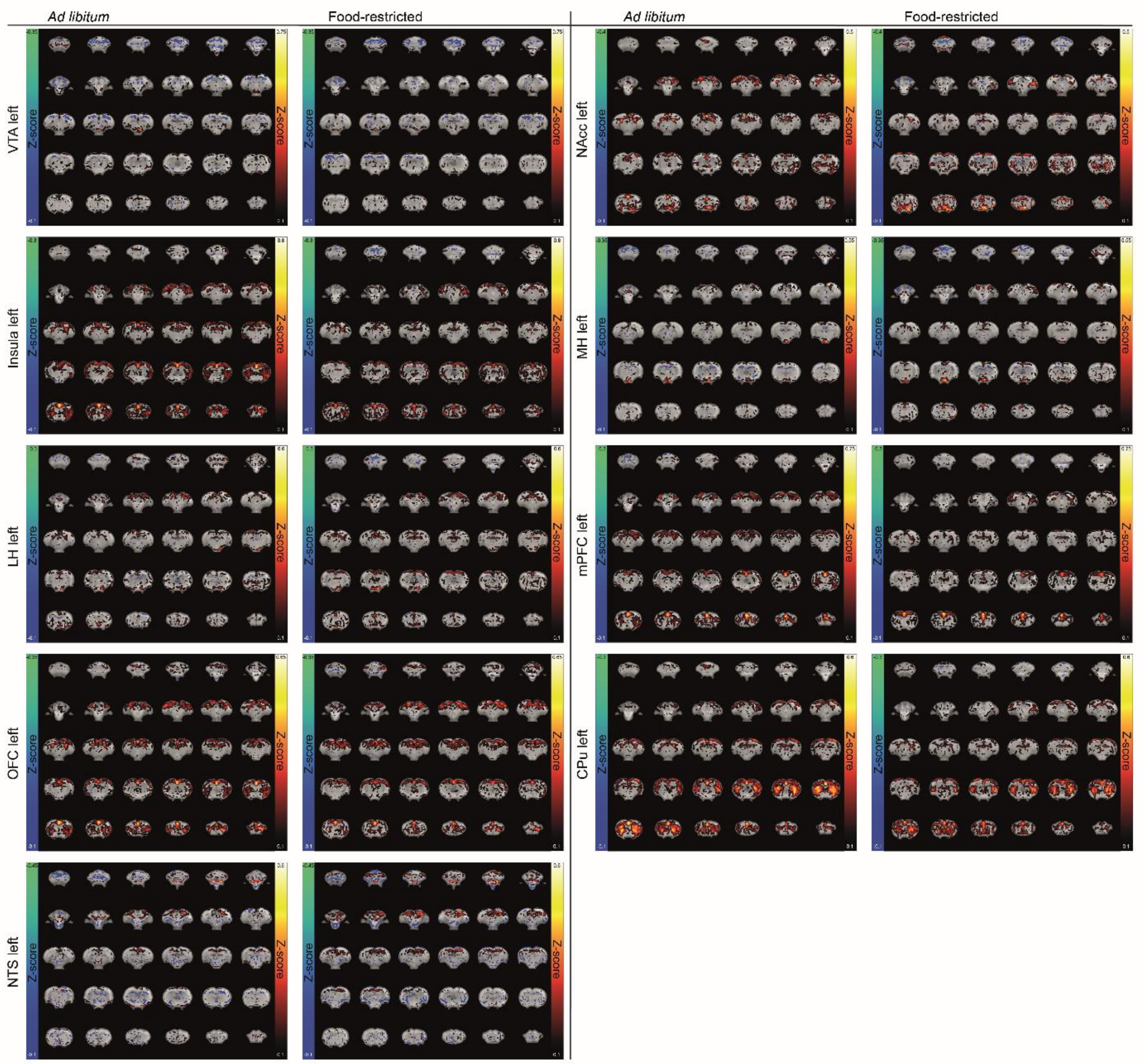

Supplementary Figure 2. Functional connectivity maps after sucrose tasting with different seed regions from *ad libitum*-fed and food-restricted rats. Mean functional connectivity maps were obtained by calculation of the Fisher-transformed z' of Pearson correlation coefficient *r* and show functional connectivity for *ad libitum*-fed (left) and food-restricted rats (right) post-sucrose tasting. Different left ROIs were used as seed regions: CPu, caudate putamen; LH, lateral hypothalamus; MH, medial hypothalamus; mPFC, medial prefrontal cortex; NAcc, nucleus accumbens; NTS, nucleus of the solitary tract; OFC, orbitofrontal cortex; VTA, ventral tegmental area.

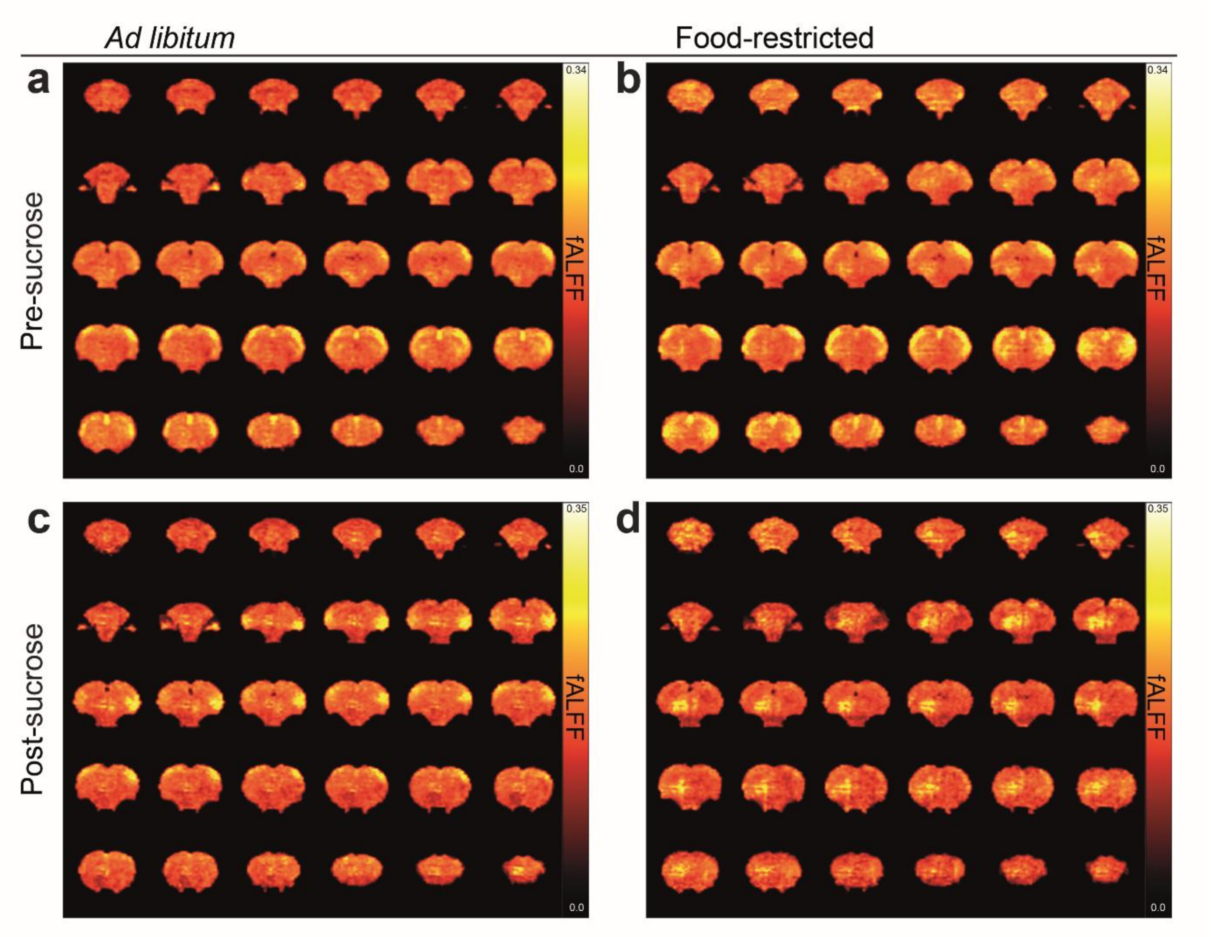

Supplementary Figure 3. Mean fractional Amplitude of Low Frequency Fluctuations (fALFF) maps in different states of energy balance. a) Whole-brain fALFF pre-sucrose tasting in *ad libitum*-fed rats. b) Whole-brain fALFF pre-sucrose tasting in food-restricted rats. c) Whole-brain fALFF post-sucrose tasting in *ad libitum*-fed rats. d) Whole-brain fALFF post-sucrose tasting in food-restricted rats.
