# Supplementary material for: Diet as connecting factor: Functional brain connectivity in relation to food intake and sucrose tasting, assessed with resting‐state functional MRI in rats
Source: J Neurosci Res. 2019 Nov 26;100(5):1182–90. doi: 10.1002/jnr.24563 (PMC9291979; doi:10.1002/jnr.24563)
Supplement: Supplementary file 3 — Transparent Peer Review Report [file JNR-100-1182-s001.docx]

Diet as connecting factor: functional brain connectivity in relation to food intake and sucrose tasting, assessed with resting-state functional MRI in rats

Dijkhuizen, Rick; Roelofs, Theresia; Straathof, Milou; Van der Toorn, Annette; Otte, Willem; Adan, Roger

_____________________________________________________________________________________

| Review timeline: | Submission date: 21/02/2019  Editorial Decision: Major Modification  Revision Received: 12/07/2019  Editorial Decision: Major Modification  Revision Received: 22/10/2019  Accepted: 10/11/2019 |  |
| --- | --- | --- |
|  |  |  |

Editor: Jerome Badaut

Reviewer: Joanes Grandjean, Francesca Mandino

_____________________________________________________________________________________

1^st^ Editorial Decision	28/03/2019

_____________________________________________________________________________________

Dear Dr Dijkhuizen:

Thank you for submitting your manuscript to the Journal of Neuroscience Research. We've now received the reviewer feedback and have appended those reviews below. As you will see, the reviewers find the question addressed to be of potential interest. Yet, they do not find the manuscript suitable for publication in its current form. Of particular note is that there is an emphasis on the fact to combine to different stimuli, which is not acceptable choice for both reviewers. Please do revise that aspect of the study.

If you feel that you can adequately address the concerns of the reviewers, you may revise and resubmit your paper within 60 days. It will require further review. Please explain in your cover letter how you have changed the present version. If you require longer than 60 days to make the revisions, please contact Dr. Eric Prager (eprager@wiley.com). You can submit your revised manuscript directly by clicking on the following link: *** PLEASE NOTE: This is a two-step process. After clicking on the link, you will be directed to a webpage to confirm. ***

https://mc.manuscriptcentral.com/jnr?URL_MASK=7339407f475d48a2a4b873d1a24d29cf

Thank you again for your submission to the Journal of Neuroscience Research; we look forward to reading your revised manuscript.

Best Wishes,

Professor Jerome Badaut

Associate Editor, Journal of Neuroscience Research

Dr Eric Prager

Editor-in-Chief, Journal of Neuroscience Research

Statistics Editor: McArthur, David

Comments to the Author:

None of the analyses are accompanied with full details as to the statistical results; only group p values (and p-values for two interactions) are shown. Kindly supply the missing information. Note that the caption for Figure 3 mentions a significant interaction without presenting any statistical finding and Figure 4 omits any mention of same. As it is clear from the concluding paragraph that this study is to help "further disentangle the complex interaction" a great deal more attention needs to be paid to formal presentation of such findings.

This journal does not accept Figure 3's and 4's "dynamite plots" (also called "plunger plots", "toiletbrush plots", or "bogbrush plots") as they are often severe distortions of the actual data distributions. When samples are small, kindly use dotplots, else use boxplots or violin plots with or without dotplot overlays.

Figure 4 notes "a visual trend" which is not presented in the Results narrative and not analyzed. Trend, however, is mentioned twice in the first paragraph of the Discussion.

Reviewer: 1

Comments to the Author

This manuscript reports on a study designed to test the hypothesis that the response to feeding behavior in satiated versus food-restricted rats is linked to alterations in brain circuits.

This is an interesting study with a reasonable hypothesis that differences in prior result are largely due to methodologic issues. Given that statement, one of the major questions of the current work is that obese rats were not used to test the main hypothesis of circuit alterations, thus it is questionable how well the circuit-level response to sucrose or gastric distention relate to the corresponding clinical cases. What is the evidence that acutely (1wk) food-deprived rats versus satiated have baseline circuits prior to food stimulus that are consistent with clinically obese people? In fact, the reported data shows that there are no baseline circuit differences between food deprived and satiated. The authors should comment on how that relates to the clinical condition. It’s possible that this absence of difference invalidates the model and/or the stimulus-induced changes in circuits.

There was no filled out Transparent Science Questionnaire.

The low numbers per group is a critical concern, especially given that data was pooled between sucrose and gastric distension groups. Yet 3 rats were excluded but it was not clear from which groups. The authors should make efforts to include more rats per group rather than pooling rats from very different stimuli conditions.

After filtering images for S:N, the authors should make it clear whether the FC data obtained from brain regions were common to all rats, or whether in areas where SN was below threshold, was the group size lower for specific regions. This may well have impacted the statistics.

Fig 1c, d should be overlayed with region-specific statistics.

For Fig 2 it’s not clear what is being shown. The assumption is that these are seed-to-whole-brain, 1-sample t test (mean) data. The voxel-based statistical contrast between groups should be presented.

Fig. 3- Plots contain too many data points- split up by region or group comparisons. It was not made clear how the intra and interhemispheric connectivity maps were calculated. Therefore it’s difficult to understand what the fc data in Fig 3a,b mean. The assumption is that it is average fc between seed region and the same hemisphere vs opposite hemisphere for intra and interhemispheric fc respectively. The authors should include the seed-to-seed interhemispheric maps as statistical contrast maps for fig. 3a,b.

Throughout the manuscript energy homeostasis is referred to, yet nowhere was this measured within the brain. This is merely a supposition based on food intake. While FC might well depend on this after all other energy-producing pathways have been exhausted, unless this can be directly measured, it should only be discussed briefly rather than being reported in the figure legends and result section.

Reviewer: 2

Comments to the Author

This manuscript aims to investigate brain changes in food-restricted status compared to fed status in Wistar rats. Functional connectivity and magnitude of neural signals are measured with rs-fMRI on food-restricted or satiated rats, before and after food-related stimulation, being this either sugar tasting, or gastric expansion. Higher functional connectivity, although non significant, was found in fed rats compared to food-restricted rats. This reversed after the stimuli. Feeding-related stimuli affected significantly functional connectivity in fed rats, no significant difference was reported for food-restricted animals. Amplitude of low-frequency signals also did not show significance.

Overall the study is well written; the authors highlight the limitations of their work, among which the small number of animals. This reviewer would like to highlight some major points:

The study is focused on feeding behaviour, based on investigating functional connectivity changes with rs-fMRI using a rat model: however, in the introduction there is a complete lack of literature regarding animal studies within the same field. The authors report several studies conducted on human participants: an overview of the background related to rodents, for this study, is highly necessary.

The authors miss to define the error bars in the results reported. Moreover, the statistical analyses reported are unclear. The reader does not clearly understand what is the statistical unit used throughout the study, on which the authors base their conclusions.

The authors use two very different types of feeding-related stimuli: sucrose tasting and gastric expansion. While the choice for the former stimulus can be guessed, there is no exhaustive explanation for the choice of the second stimulus, nor for the need to have two separate stimuli.

The authors merge data from sucrose and gastric expansion for the analysis of whole brain functional connectivity maps. The reviewer is not in line with this choice, as treating different stimuli as eliciting the same response brings a confounding factor in the analysis: sucrose tasting and gastric expansion ought to elicit different brain and physiological responses (if not, supporting evidence should be provided). This, taken together with the small sample number per group, and the non-significance of some of the results, leads the reviewer to suggest a revision of the study design. Given the lack of justification for the use of two different stimuli, the reviewer suggests to apply the same stimulus to all animals. Upon exhaustive explanation for the use of two different stimuli instead, the authors could, for example, apply both stimuli onto all animals, consecutively. It would be interesting to study the different responses caused by the usage of different stimulation sources.

Overall, the design of the study should be substantially revised as it currently does not bring convincing evidence for its role in advancing the field of feeding behaviour in rodents.

___________________________________________________________________________________

Authors’ Response	12/07/2019

_____________________________________________________________________________________

Dear Prof. Badaut and Dr. Prager,

Thank you for reviewing our manuscript. We also thank the reviewers for their critical assessment of our paper. We have revised our manuscript based on their constructive feedback. Importantly, as you have suggested, we have refrained from pooling data from two stimuli groups in the revised paper. We feel that our manuscript has significantly approved and hope that the revised version meets the standard of the Journal of Neuroscience Research.

Our specific responses to the reviewers’ points are outlined below.

We look forward to your response.

Thank you again.

Best regards, on behalf of all authors,

Rick Dijkhuizen

Specific responses to reviewers’ points:

Statistics Editor: McArthur, David

Comments to the Author:

None of the analyses are accompanied with full details as to the statistical results; only group p values (and p-values for two interactions) are shown. Kindly supply the missing information.

Our response: We thank the editor for this notion and have now supplied the estimates, 95% confidence intervals, t-values with degrees of freedom, and p-values with the significant findings and trends.

Note that the caption for Figure 3 mentions a significant interaction without presenting any statistical finding and Figure 4 omits any mention of same.

Our response: We have performed new analyses and the revised Figure 3 now shows a trend whereas a significant effect was found for data shown in Figure 4. We have now mentioned this in the revised figure legends.

As it is clear from the concluding paragraph that this study is to help "further disentangle the complex interaction" a great deal more attention needs to be paid to formal presentation of such findings.

Our response: We hope that the revisions and adjustments that we have made in response to the reviewers’ comments, as outlined below, now provide the requested improvement in the formal presentation of our findings.

This journal does not accept Figure 3's and 4's "dynamite plots" (also called "plunger plots", "toiletbrush plots", or "bogbrush plots") as they are often severe distortions of the actual data distributions. When samples are small, kindly use dotplots, else use boxplots or violin plots with or without dotplot overlays.

Our response: We agree with the reviewer and have changed Figures 3 and 4 to boxplots.

Figure 4 notes "a visual trend" which is not presented in the Results narrative and not analyzed. Trend, however, is mentioned twice in the first paragraph of the Discussion.

Our response: We have performed new analyses and the revised Figure 4 now shows a significant effect. We have mentioned this in the revised figure legend and the revised Results and Discussion sections.

Reviewer: 1

Comments to the Author

This manuscript reports on a study designed to test the hypothesis that the response to feeding behavior in satiated versus food-restricted rats is linked to alterations in brain circuits.

This is an interesting study with a reasonable hypothesis that differences in prior result are largely due to methodologic issues. Given that statement, one of the major questions of the current work is that obese rats were not used to test the main hypothesis of circuit alterations, thus it is questionable how well the circuit-level response to sucrose or gastric distention relate to the corresponding clinical cases. What is the evidence that acutely (1wk) food-deprived rats versus satiated have baseline circuits prior to food stimulus that are consistent with clinically obese people? In fact, the reported data shows that there are no baseline circuit differences between food deprived and satiated. The authors should comment on how that relates to the clinical condition. It’s possible that this absence of difference invalidates the model and/or the stimulus-induced changes in circuits.

Our response: We thank the reviewer for raising this point. Our study, however, did not aim to assess differences between obese and lean rats (or individuals), but between satiated and hungry rats. We wanted to investigate if status of current energy balance, thus being hungry or satiated, affects the organisation of neural networks. Our data suggest that there is no significant difference. It may be that obesity does alter neural network organisation, however, that falls beyond the scope of the current study. To take away any impression that our study was designed to elucidate mechanisms underlying obesity, we now place our study in a broader context by mentioning in the revised Introduction that disturbed regulation of feeding behaviour and energy balance can lead to eating disorders as well as obesity (page 4, lines 69-70). We agree with the reviewer that it would be very interesting to repeat this study in obese and lean rats to assess the differences in neural network organisation, as we now mention in the revised Discussion (page 16, lines 379-381). Our neutral findings do not invalidate the model per se. We have used this same model in a previous study to assess responses in neural activity to taste and gastric distention and found significant differences. These data are, however, not published yet.

Whether being hungry or satiated changes the organisation of neural networks translates to the clinic in such a way that the question remains the same. It could very well be that in humans current status of energy balance also doesn’t affect neural network organisation, but that obesity might affect this.

There was no filled out Transparent Science Questionnaire.

Our response. Our apologies. We have now filled out this quesionnaire

The low numbers per group is a critical concern, especially given that data was pooled between sucrose and gastric distension groups. Yet 3 rats were excluded but it was not clear from which groups. The authors should make efforts to include more rats per group rather than pooling rats from very different stimuli conditions.

Our response: We have carefully considered this concern of the reviewer and agree that the relatively low numbers of rats are a limitation of our study. We now mention this explicitly in the revised Discussion (page 15, lines 359-360; page 16, lines 366-368). Unfortunately, because of logistic reasons (finished contract of the first author, no remaining project budget, etc.) we are unable to perform additional experiments.

To take away the concern of pooling of data from very different stimuli conditions, we have excluded the gastric distention data and exclusively focused our post-stimulus analyses on the effects of sucrose tasting, as also suggested by Reviewer 2. No data were excluded for the pre-stimulus analyses. This led to the following sample sizes: ad libitum-fed group pre-sucrose, n=7; food-restricted group pre-sucrose, n=6; ad libitum-fed group post-sucrose, n=3; and food-restricted group post-sucrose, n=3. This has been indicated throughout the revised manuscript. Furthermore, in the revised Results section we now state specifically from which groups animals were excluded due to MRI artefacts (page 12, lines 281-284).

We understand that the low sample size in the post-sucrose groups is a limitation. Therefore, we have interpreted and discussed these results with extra caution throughout the revised manuscript.

After filtering images for S:N, the authors should make it clear whether the FC data obtained from brain regions were common to all rats, or whether in areas where SN was below threshold, was the group size lower for specific regions. This may well have impacted the statistics.

Our response: We have used a tSNR-mask to filter out voxels with low (<10) tSNR. In some cases, this led to exclusion of individual voxels within regions, but never to exclusion of entire brain regions or rats. In other words, group sizes remained unchanged by this procedure. We have clarified this in the revised Methods section (page 10, lines 222-223).

Fig 1c, d should be overlayed with region-specific statistics.

Our response: We appreciate the reviewer’s comment, but it is rather unusual to overlay these kinds of functional connectivity matrices with region-specific statistics. The color-bar on the right of each matrix encodes the functional connectivity values. In our opinion the matrices would become unclear if every square would contain a color and a number.

For Fig 2 it’s not clear what is being shown. The assumption is that these are seed-to-whole-brain, 1-sample t test (mean) data. The voxel-based statistical contrast between groups should be presented.

Our response: We apologize this hasn’t been clear. Figure 2 shows seed-to-whole-brain maps of functional connectivity, expressed by the Fisher-transformed z’ of Pearson correlation coefficient r (see page 10, lines 223-228). These maps are calculated with standard software packages provided by FSL. The different regions of interest (left-sided for Figure 2 and right-sided for Supplementary Figure 1) are used as seed regions for the different maps.

Fig. 3- Plots contain too many data points- split up by region or group comparisons. It was not made clear how the intra and interhemispheric connectivity maps were calculated. Therefore it’s difficult to understand what the fc data in Fig 3a,b mean. The assumption is that it is average fc between seed region and the same hemisphere vs opposite hemisphere for intra and interhemispheric fc respectively. The authors should include the seed-to-seed interhemispheric maps as statistical contrast maps for fig. 3a,b.

Our response: Figure 3 displays functional connectivities between homologous regions of interest in both hemispheres (interhemispheric functional connectivity in Figure 3a) or between regions of interest within hemispheres (intrahemispheric functional connectivity in Figure 3b). This has now been clarified in the revised Methods section of the manuscript (page 10, lines 235-238).

Throughout the manuscript energy homeostasis is referred to, yet nowhere was this measured within the brain. This is merely a supposition based on food intake. While FC might well depend on this after all other energy-producing pathways have been exhausted, unless this can be directly measured, it should only be discussed briefly rather than being reported in the figure legends and result section.

Our response: We thank the reviewer for this comment. We have now limited our statements and discussion on energy homeostasis, or explained the context more explicitly, throughout the revised manuscript. We refrained from reference to energy homeostasis in the revised figure legends and Results section.

Reviewer: 2

Comments to the Author

This manuscript aims to investigate brain changes in food-restricted status compared to fed status in Wistar rats. Functional connectivity and magnitude of neural signals are measured with rs-fMRI on food-restricted or satiated rats, before and after food-related stimulation, being this either sugar tasting, or gastric expansion. Higher functional connectivity, although non significant, was found in fed rats compared to food-restricted rats. This reversed after the stimuli. Feeding-related stimuli affected significantly functional connectivity in fed rats, no significant difference was reported for food-restricted animals. Amplitude of low-frequency signals also did not show significance.

Overall the study is well written; the authors highlight the limitations of their work, among which the small number of animals. This reviewer would like to highlight some major points:

1. The study is focused on feeding behaviour, based on investigating functional connectivity changes with rs-fMRI using a rat model: however, in the introduction there is a complete lack of literature regarding animal studies within the same field. The authors report several studies conducted on human participants: an overview of the background related to rodents, for this study, is highly necessary.

Our response: We thank the reviewer for making this point. We are not aware of any literature reporting on resting state-fMRI in rodents in the field of food intake or tasting, as mentioned in the revised Introduction section (page 5, lines 110-112). We did, however, added reference to two papers by Min and colleagues (2011a, b) who assessed changes in neural activity in response to gastric infusion of nutrients or gastric distention in rats (page 5-6, lines 112-114).

2. The authors miss to define the error bars in the results reported.

Moreover, the statistical analyses reported are unclear. The reader does not clearly understand what is the statistical unit used throughout the study, on which the authors base their conclusions.

Our response: We thank the reviewer for this notion. All error bars represented SD. However, since we changed the graphs to boxplots, following the advice of the Statistics Editor, the bars now represent minimum to maximum value. We have mentioned this in the revised figure legends.

To clarify our statistics, we now report p-values, estimates, confidence intervals, and t-values with degrees of freedom, for all significant effects and trends.

3. The authors use two very different types of feeding-related stimuli: sucrose tasting and gastric expansion. While the choice for the former stimulus can be guessed, there is no exhaustive explanation for the choice of the second stimulus, nor for the need to have two separate stimuli.

Our response: We agree with the reviewer. This point has also been raised by the other reviewer and by the editors. Therefore we have decided to exclude the gastric distention data and only focus on the effects of sucrose tasting.

4. The authors merge data from sucrose and gastric expansion for the analysis of whole brain functional connectivity maps. The reviewer is not in line with this choice, as treating different stimuli as eliciting the same response brings a confounding factor in the analysis: sucrose tasting and gastric expansion ought to elicit different brain and physiological responses (if not, supporting evidence should be provided). This, taken together with the small sample number per group, and the non-significance of some of the results, leads the reviewer to suggest a revision of the study design. Given the lack of justification for the use of two different stimuli, the reviewer suggests to apply the same stimulus to all animals. Upon exhaustive explanation for the use of two different stimuli instead, the authors could, for example, apply both stimuli onto all animals, consecutively. It would be interesting to study the different responses caused by the usage of different stimulation sources.

Our response: The reviewer is right and we thank him or her for the critical feedback. To take away the concern of pooling of data from very different stimuli conditions, we have excluded the gastric distention data and exclusively focused our post-stimulus analyses on the effects of sucrose tasting, as also suggested by Reviewer 1. No data were excluded for the pre-stimulus analyses. This led to the following sample sizes: ad libitum-fed group pre-sucrose, n=7; food-restricted group pre-sucrose, n=6; ad libitum-fed group post-sucrose, n=3; and food-restricted group post-sucrose, n=3. We understand that the low sample size in the post-sucrose groups is a limitation. Therefore, we have interpreted and discussed these results with extra caution throughout the revised manuscript.

Unfortunately, because of logistic reasons (finished contract of the first author, no remaining project budget, etc.) we are unable to perform additional experiments.

Overall, the design of the study should be substantially revised as it currently does not bring convincing evidence for its role in advancing the field of feeding behaviour in rodents.

Our response: We feel that our paper has significantly improved based on the revisions and adjustments that we have made in response to the reviewers’ comments, and we hope that the revised manuscript now meets the criteria for publication in the Journal of Neuroscience Research.

_____________________________________________________________________________________

2^nd^ Editorial Decision	28/08/2019

_____________________________________________________________________________________

Dear Dr Dijkhuizen:

Thank you for submitting your manuscript to the Journal of Neuroscience Research. We've now received the reviewer feedback and have appended those reviews below. While the authors have addressed most of the previous comments from the first round of reviewers, both reviewers and the statistical editor still underlined some issues with the stats, which are critical in the network analysis after fMRI. We believe that the paper will be suitable for publication, but only after the statistics are fully addressed.

If you feel that you can adequately address the concerns of the reviewers, you may revise and resubmit your paper within 60 days. It will require further review. Please explain in your cover letter how you have changed the present version. If you require longer than 60 days to make the revisions, please contact Dr. Eric Prager (eprager@wiley.com). You can submit your revised manuscript directly by clicking on the following link: *** PLEASE NOTE: This is a two-step process. After clicking on the link, you will be directed to a webpage to confirm. ***

https://mc.manuscriptcentral.com/jnr?URL_MASK=f28b813d340e40be84a986e7e249326c

Thank you again for your submission to the Journal of Neuroscience Research; we look forward to reading your revised manuscript.

Best Wishes,

Professor Jerome Badaut

Associate Editor, Journal of Neuroscience Research

Dr Eric Prager

Editor-in-Chief, Journal of Neuroscience Research

Statistics Editor: McArthur, David

Comments to the Author:

The statistical issues raised by the other reviewers must be addressed.

Additional comments: Results paragraph 2 presents a result for the statistical interaction of group and time, but nothing comparable is shown in the paragraph that follows even though it would seem to be important there.

Discussion indicates that their analysis "revealed a decrease in fALFF in response to sucrose tasting." However, in the Results section this is qualified by "probably reflects" and "might be related", which are indicators of hesitation in making the claim. Which is right?

Statistical results shown in Results are repeated in full unnecessarily in the figure captions.

Minor point: the decimal precision of various p-values is not consistent. Kindly choose one level (say three digits) and use throughout.

Reviewer: 1

Comments to the Author

The manuscript by Roelofs et al. describes a whole-brain functional imaging experiment in rats under either food restrictions or ad libidum food, and following sugar intake. The authors use network analysis and fractional amplitude of low-frequency fluctuations, and reports mostly decreased inter-hemispheric correlations following sugar intake, together with group : time interaction effects in the fractional amplitude of low-frequency fluctuation.

Despite significant improvement in the statistic presentation, the authors have brushed off an important point made by reviewer #2 regarding the statistical unit. In the revamped manuscript, the authors declare between 147 and 160 degrees of freedom (DOF), from 16 rats (minus some exclusions) in their tests. Only 16 independent samples were recorded, hence it appears the authors violated the assumption of independence in their test.

Estimating DOF in a linear mixed model is tricky. Some argue this exercise is futile (https://stat.ethz.ch/pipermail/r-help/2006-May/094765.html). However, if the stated DOFs are a correct depiction of the tests carried out, this would indicate that the statistical unit is not ‘animal’, but ‘ROI X imaging session”. Indeed, 160 DOF/ 9 ROI gives 17.7, more or less the number of animals (including the two time points which are also not independent from each other).

In theory, a linear mixed model package such as nlme should account for the DOF if ‘animal’ was listed as a random effect, e.g. if 9 samples are drawn from the same individual, this would only count as one DOF. However, the authors only mention ‘group’ and ‘time’ as factors (fixed effects?) in the method but do not describe random effects. Moreover, there is no mention of ‘ROI’ either as a fixed or a random effect. In principle, packages such as nlme shouldn’t be able to operate without random effects, so it is unclear where the error occurred. If random effects were used properly, there shouldn’t be 147-160 DOF, but if the DOF were misreported in the manuscript despite running the tests correctly, t-values of 1.7826 wouldn’t give a p-value of 0.07 but rather 0.09. A marginal difference.

This confusion over the statistical unit is important to clarify. Statistical units define from which populations the samples are drawn, and hence to what population the results can be generalized to. In cases where ‘animal’ is the statistical unit, this is simple, the population is all the Wistar rats in the world. In the case of ‘ROI X animal’, it is less clear if the results should be generalized to a larger population of rats, or a larger population of ‘ROI’. There is, of course, little interest to generalize to a larger population of ROI. If they did so, they could only conclude that if they included additional ROIs to their 9 ROIs, there is a high probability that they would display the same effect in these animals, and these animals only.

Finally, if the authors perform one statistical test merging several ROIs per analysis, what is being corrected for multiple comparisons, as stated in the method section? Moreover, nlme allows fitting a model, but AFAIK does not supply p-values. There are a number of methods to obtain p-values from linear mixed models, some of which provide F values instead of T values (https://www.ssc.wisc.edu/sscc/pubs/MM/MM_TestEffects.html). It isn’t clear how this was done presently, and why t-values are presented.

The authors should clarify the statistics section as there is a lot of confusion remaining. They should clearly define their statistical unit, verify the DOFs, and state clearly what are the fixed and random effects. If the authors use something else than ‘animal’ as a statistical unit, this should be justified and the discussion should match the scope of that decision. Finally, this reviewer is fully in favour of moving beyond p-value inference (https://www.nature.com/articles/s41592-019-0470-3). This reviewer would prefer an emphasis on effect size (+ 95%CI) and a clearer and honest representation of the data, rather than possibly incorrect statistics.

Reviewer: 2

Comments to the Author

This manuscript aims to investigate the relation between food intake and brain connectivity in the rat brain.

This reviewer appreciates the effort of the authors to ameliorate their manuscript and to address reviewer's comments. However, this reviewer is not fully convinced on how these results can help shed a light onto the relationship of brain functional connectivity and food intake/sucrose tasting.

This reviewer is still skeptical about the statistics reported. For example, in-text lines 294-300 describe matrices of FC, yet the authors report (in the same text) one single p-value analyses (p = 0.57 and 0.69). What do these p values refer to? These two p-values 0.57 and 0.69 are found again in the caption of Figure 4, but that figure reference is not mentioned in text 294-300. Overall, a more clear overview of what the results mean, and which figure they describe, should be carried out.

Moreover, more clarity on what the p values represent, i.e. the meaning of the statistical unit adopted (e.g. animal or edge) would be crucial for a better understanding of the results.

Additionally, the reviewer's suggestion to separate sugar-tasting and gastric-expansion animals did not necessarily mean to have a complete exclusion of the gastric-expansion data set, being this around half of the total experimental data. Given the results provided so far, not fully convincing and relatively of minor relevance, it would be interesting to maybe see individual analysis of each of the two groups, treating them as two separate approaches to investigate a common aspect of brain connectivity.

As minor consideration, the figures quality is poor and labels can barely be readable (e.g. values on the color bars in Fig 2).

___________________________________________________________________________________

Authors’ Response	22/10/2019

_____________________________________________________________________________________

Dear Editors,

We thank you and the reviewers for reviewing our manuscript. The reviewers’ comments were helpful and we appreciate the opportunity given to us to revise our manuscript accordingly.

Our responses to the reviewers’ comments, including explanation of our revisions, are outlined below.

Statistics Editor:

The statistical issues raised by the other reviewers must be addressed.

Our response: This is addressed below.

Additional comments: Results paragraph 2 presents a result for the statistical interaction of group and time, but nothing comparable is shown in the paragraph that follows even though it would seem to be important there.

Our response: We agree that this part should have been integrated in the third paragraph and moved it to this section.

Discussion indicates that their analysis "revealed a decrease in fALFF in response to sucrose tasting." However, in the Results section this is qualified by "probably reflects" and "might be related", which are indicators of hesitation in making the claim. Which is right?

Our response: We thank the Reviewer for pointing this out and downplayed our statement by replacing ‘revealed a decrease’ by ‘suggested a decrease’ (Discussion, first paragraph).

Statistical results shown in Results are repeated in full unnecessarily in the figure captions.

Our response: We have deleted the full repeats of the statistical results from the figure legends.

Minor point: the decimal precision of various p-values is not consistent. Kindly choose one level (say three digits) and use throughout.

Our response: We have chosen a three-digits-level of precision throughout the revised manuscript.

Reviewer 1:

Despite significant improvement in the statistic presentation, the authors have brushed off an important point made by reviewer #2 regarding the statistical unit. In the revamped manuscript, the authors declare between 147 and 160 degrees of freedom (DOF), from 16 rats (minus some exclusions) in their tests. Only 16 independent samples were recorded, hence it appears the authors violated the assumption of independence in their test.

Estimating DOF in a linear mixed model is tricky. Some argue this exercise is futile (https://stat.ethz.ch/pipermail/r-help/2006-May/094765.html). However, if the stated DOFs are a correct depiction of the tests carried out, this would indicate that the statistical unit is not ‘animal’, but ‘ROI X imaging session”. Indeed, 160 DOF/ 9 ROI gives 17.7, more or less the number of animals (including the two time points which are also not independent from each other).

In theory, a linear mixed model package such as nlme should account for the DOF if ‘animal’ was listed as a random effect, e.g. if 9 samples are drawn from the same individual, this would only count as one DOF. However, the authors only mention ‘group’ and ‘time’ as factors (fixed effects?) in the method but do not describe random effects. Moreover, there is no mention of ‘ROI’ either as a fixed or a random effect. In principle, packages such as nlme shouldn’t be able to operate without random effects, so it is unclear where the error occurred. If random effects were used properly, there shouldn’t be 147-160 DOF, but if the DOF were misreported in the manuscript despite running the tests correctly, t-values of 1.7826 wouldn’t give a p-value of 0.07 but rather 0.09. A marginal difference.

This confusion over the statistical unit is important to clarify. Statistical units define from which populations the samples are drawn, and hence to what population the results can be generalized to. In cases where ‘animal’ is the statistical unit, this is simple, the population is all the Wistar rats in the world. In the case of ‘ROI X animal’, it is less clear if the results should be generalized to a larger population of rats, or a larger population of ‘ROI’. There is, of course, little interest to generalize to a larger population of ROI. If they did so, they could only conclude that if they included additional ROIs to their 9 ROIs, there is a high probability that they would display the same effect in these animals, and these animals only.

Finally, if the authors perform one statistical test merging several ROIs per analysis, what is being corrected for multiple comparisons, as stated in the method section? Moreover, nlme allows fitting a model, but AFAIK does not supply p-values. There are a number of methods to obtain p-values from linear mixed models, some of which provide F values instead of T values (https://www.ssc.wisc.edu/sscc/pubs/MM/MM_TestEffects.html). It isn’t clear how this was done presently, and why t-values are presented.

The authors should clarify the statistics section as there is a lot of confusion remaining. They should clearly define their statistical unit, verify the DOFs, and state clearly what are the fixed and random effects. If the authors use something else than ‘animal’ as a statistical unit, this should be justified and the discussion should match the scope of that decision. Finally, this reviewer is fully in favour of moving beyond p-value inference (https://www.nature.com/articles/s41592-019-0470-3). This reviewer would prefer an emphasis on effect size (+ 95%CI) and a clearer and honest representation of the data, rather than possibly incorrect statistics.

Our response: We feel that these statistical issues, raised by Reviewer 1, are the consequence of the limited explanation of our statistical approach in the previous version of our manuscript. We apologize for the confusion that arose from this. For example, we did correct for the factors ‘rat’ and ‘ROI’ in our original analyses. However, this was not clearly explained in the Methods section. In the revised manuscript, we have clarified our statistical approach more carefully in the Methods section (see section ‘Resting-state fMRI data analysis – ROI analyses’) and throughout the Results section (by removing details which may have suggested a classical ANOVA approach). We have removed DOF and t-values, since this is problematic for mixed-models, as correctly indicated by the Reviewer. We now mention the 95% confidence intervals, as suggested by the Reviewer. Furthermore, we have generated a GitHub link, to which we refer in the Methods section, providing the syntax used as well as example scripts.

Reviewer 2:

This manuscript aims to investigate the relation between food intake and brain connectivity in the rat brain.

This reviewer appreciates the effort of the authors to ameliorate their manuscript and to address reviewer's comments. However, this reviewer is not fully convinced on how these results can help shed a light onto the relationship of brain functional connectivity and food intake/sucrose tasting.

This reviewer is still skeptical about the statistics reported. For example, in-text lines 294-300 describe matrices of FC, yet the authors report (in the same text) one single p-value analyses (p = 0.57 and 0.69). What do these p values refer to? These two p-values 0.57 and 0.69 are found again in the caption of Figure 4, but that figure reference is not mentioned in text 294-300. Overall, a more clear overview of what the results mean, and which figure they describe, should be carried out.

Moreover, more clarity on what the p values represent, i.e. the meaning of the statistical unit adopted (e.g. animal or edge) would be crucial for a better understanding of the results.

Our response: We thank Reviewer 2 for pointing this out. In the revised manuscript, we have clarified our statistical approach and results in the Methods and Results sections, respectively. Furthermore, we have provided a GitHub link with the syntax used as well as example scripts.

Additionally, the reviewer's suggestion to separate sugar-tasting and gastric-expansion animals did not necessarily mean to have a complete exclusion of the gastric-expansion data set, being this around half of the total experimental data. Given the results provided so far, not fully convincing and relatively of minor relevance, it would be interesting to maybe see individual analysis of each of the two groups, treating them as two separate approaches to investigate a common aspect of brain connectivity.

Our response: We agree that it would be interesting to re-include the gastric-expansion dataset and perform a separate analysis on the individual groups. However, unfortunately, due to lack of personnel (the first author who has executed the experiments and analyses has left our department), we will not be able to execute this in a timely manner.

As minor consideration, the figures quality is poor and labels can barely be readable (e.g. values on the color bars in Fig 2).

Our response: We have uploaded the figures as high-resolution images. Probably, the converted PDF proofs contained lower quality/resolution versions of the figures.
